# SVachra: a tool to identify genomic structural variation in mate pair sequencing data containing inward and outward facing reads

**DOI:** 10.1186/s12864-017-4021-y

**Published:** 2017-10-03

**Authors:** Oliver A. Hampton, Adam C. English, Mark Wang, William J. Salerno, Yue Liu, Donna M. Muzny, Yi Han, David A. Wheeler, Kim C. Worley, James R. Lupski, Richard A. Gibbs

**Affiliations:** 1 Human Genome Sequencing Center, Baylor College of Medicine, 1 Baylor Plaza, Houston, TX 77030 USA; 2 Department of Molecular and Human Genetics, Baylor College of Medicine, 1 Baylor Plaza, Houston, TX 77030 USA; 3 Department of Pediatrics, Baylor College of Medicine, 1 Baylor Plaza, Houston, TX 77030 USA; 4 Texas Children’s Hospital, 6621 Fanin Street, Houston, TX 77030 USA

**Keywords:** Structural variation, Breakpoint discovery, Mate pair sequencing

## Abstract

**Background:**

Characterization of genomic structural variation (SV) is essential to expanding the research and clinical applications of genome sequencing. Reliance upon short DNA fragment paired end sequencing has yielded a wealth of single nucleotide variants and internal sequencing read insertions-deletions, at the cost of limited SV detection. Multi-kilobase DNA fragment mate pair sequencing has supplemented the void in SV detection, but introduced new analytic challenges requiring SV detection tools specifically designed for mate pair sequencing data. Here, we introduce SVachra – Structural Variation Assessment of CHRomosomal Aberrations, a breakpoint calling program that identifies large insertions-deletions, inversions, inter- and intra-chromosomal translocations utilizing both inward and outward facing read types generated by mate pair sequencing.

**Results:**

We demonstrate SVachra’s utility by executing the program on large-insert (Illumina Nextera) mate pair sequencing data from the personal genome of a single subject (HS1011). An additional data set of long-read (Pacific BioSciences RSII) was also generated to validate SV calls from SVachra and other comparison SV calling programs. SVachra exhibited the highest validation rate and reported the widest distribution of SV types and size ranges when compared to other SV callers.

**Conclusions:**

SVachra is a highly specific breakpoint calling program that exhibits a more unbiased SV detection methodology than other callers.

**Electronic supplementary material:**

The online version of this article (doi:10.1186/s12864-017-4021-y) contains supplementary material, which is available to authorized users.

## Background

The identification of genomic SV is an integral part of understanding human genetic diversity, gene and genome variation, evolution, and disease etiology. Numerous genetic diseases, most notably cancer, have been associated with genomic SV of somatic cells [1] whilst germline genomic rearrangements cause a wide range of genomic disorders by diverse structural variation mutagenesis mechanisms [2, 3]. Historically, clone-based methods, such as end sequence profiling of Bacterial Artificial Chromosome and fosmid clones [4, 5] have provided paired-end sequencing of DNA fragments containing aberrant junctions, necessary to identify genomic structural rearrangements. Unfortunately, these clone-based methods proved too laborious, time consuming, and costly for routine chromosome aberration analyses. Alternatively, the adoption of massively parallel sequencing delivering hundreds of millions of short fragment paired-end reads has accelerated the study of genomics. While the quantity of reads produced by massively parallel sequencing is impressive, the standard paired-end sequencing libraries have sacrificed fragment sizes compared to clone-based methods, thereby limiting the detection of large complex genomic SV [6]. To alleviate the shortcomings of small fragment paired-end libraries, multi-kilobase mate pair and Nextera Tagmentation sequencing libraries have been introduced. For these long-range mate pair libraries, fragments (usually on the order of 3-10Kb) are isolated, end labeled with biotin and circularized. The circularized molecules are sheared and the library is enriched for biotin labeled junction fragments. Sequencing of these biotinylated fragments generates ‘outward-facing’ paired reads, meaning they align to the reference sequence in an outward facing direction from each other and at a distance in line with the selected long-range fragment size. The main restriction of such sequencing libraries is the contamination of inward facing reads, unbiotinylated fragments which map in an opposite orientation and smaller fragment size (usually between 200 and 300 bp) that confound the calling of chromosomal rearrangements by introducing contradictory discordant read information.

To improve the utility of multi-kilobase mate pair and Nextera Tagmentation sequencing libraries for genomic SV detection, we introduce a new freely accessible program called SVachra (Structural Variation Assessment of CHRomosomal Aberrations) that uses discordant mate pair reads consisting of both inward and outward facing read types. The SVachra program calculates the distributions of the inward and outward facing mate pair types and applies independent clustering of the inward and outward facing discordant mapped reads to call chromosomal structural variants. Both inward and outward facing reads contribute to SV calling, thereby improving the accuracy of the breakpoint location. SVachra identifies large insertions-deletions, inversions, inter- and intra-chromosomal translocations. Compared to other SV prediction tools that utilize mate pair mapping data; such as BreakDancer [7], SVDetect [8], and others (see review [9]), the utility and novelty of SVachra consists in its ability to (i) automatically segregate outward and inward facing reads based on K-Means clustering of fragment size distributions without a priori sequencing library information, (ii) independently cluster and merge discordant outward and inward facing reads to more accurately predict breakpoint locations, (iii) screen out user-defined segments of the reference for breakpoint consideration, such as centromeric and telomeric regions responsible for high false positive rates and heavy computation, and (iv) create various output file formats for visual assessment of SV and downstream processes, such as breakpoint spanning primer design.

### Methods

**Fig. 1:**
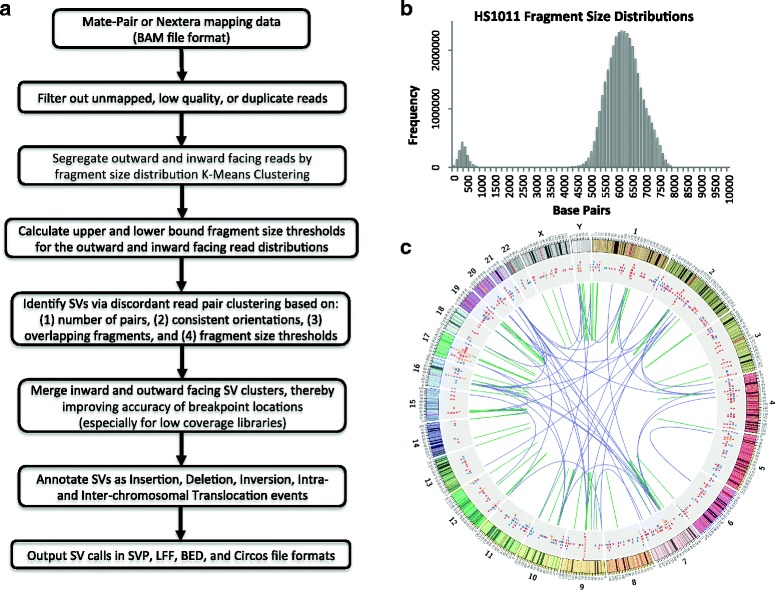
Overview of SVachra and sample output. **a** Workflow schematic. **b** Insert size distribution of the HS1011 Nextera mate pair sequencing library with ~300 bp inward facing read fragments and ~6.5 Kb outward facing read fragments. **c** Circos [10] visualization of putative SVs detected in the HS1011 Nextera sequencing library with tiles: insertions (blue), deletions (red) and inversions (orange), and links: intra-chromosomal (green) and inter-chromosomal rearrangements (purple)

SVachra takes a .bam file of mapped mate pair reads and filters out unmapped, duplicate, and low quality reads based on a user defined minimum mapping quality parameter. Mapping quality is based on the supplied .bam file MAPQ value that equals -10log_10_ times the probability{that the mapping position is wrong}, rounded to the nearest integer (default = 0). In addition, the user may select the “-u” option that will consider only uniquely mapped reads during computation. Finally, the user may supply a screen out .bed file that will filter reads based on overlap with undesirable reference segments such as centromeres and telomeres that are responsible for false positive structural variant calls. From the remaining high quality mapped reads, SVachra calculates the inward and outward facing fragment distributions. Based on the fragment size distributions, SVachra segregates paired reads using K-Means clustering; classifying paired reads as either outward or inward facing, and calculating the upper and lower bound fragment size thresholds for the inward and outward facing read sets. Discordant read pairs, those that map with anomalous order, orientation, and/or fragment size, are utilized for putative SV calling. Discordant read pairs and/or split read alignments are clustered based on matching orientation and overlapping fragments that span the same putative breakpoint. The minimum number of breakpoint spanning read pairs or split read alignments contributing to any given cluster is a user-defined parameter (default = 2). The allowed spanning overlap distance for clustering any two paired alignment sets is constrained to two times the maximum fragment size of the appropriate read distribution, i.e. the inward or outward fragment size thresholds. Given the fragment size thresholds, there is a possibility that more than one inward or outward cluster could be generated for any single chromosomal aberration. Such an occurrence would manifest as overlapping clusters with identical read pair orientations and represent a redundant structural variant call. Cluster quality control filtering may be selected using the “-s” option that flags (and reports) redundant clusters and retains only the highest scoring cluster in an overlapping set for subsequent processing. Putative breakpoints called independently by both outward and inward facing clusters are merged, improving the accuracy of those breakpoint locations, especially in cases of low coverage sequencing data sets. Clusters are annotated into independent chromosomal aberrations and classified as insertions or deletions (indels), inversions, or translocations. Sizes of indels are calculated from the average discordant distance disparity from the expected distribution mean. Each putative SV call reports a score based on the number of contributing spanning read pairs and split read alignments for that rearrangement. And while both inward and outward facing reads types inform the assessment of structural variants, reported SVs are orientated to the inward facing read orientations allowing easy comparison and integration of SV calls with the more common paired end sequencing data. The SVachra programmatic workflow is outlined in Fig. 1a.

SVachra provides numerous outputs for subsequent SV analysis; including: binned fragment lengths for plotting the mate pair insert distributions, lists of annotated SV calls in both .svp and .lff formats (see Additional file 1: S2 for output format definitions), .bed file of SV calls for intersection analysis, and Circos [10] input files for SV visualization. Two Circos input files are provided: a Circos link input file contains the SVachra chromosomal aberrations for the inter-chromosomal (purple) and intra-chromosomal (green) translocations, and a Circos tile input file contains the deletions (red), insertions (blue), and inversions (orange).

## Results and discussion

SVachra was evaluated on a 6.2Kb average insert Nextera Tagmentation mate pair library (yielding over 90 million reads and 3X genomic sequence coverage) of the HS1011 human genome that contains a causative single nucleotide variant for an autosomal recessive Charcot-Marie-Tooth neuropathy [2, 11, 12]. The binned fragment lengths for the HS1011 Nextera mate pair sequencing library is shown in Fig. 1b and highlights the two separate insert size distributions, the inward facing reads with an average fragment length of ~450 bp and outward facing reads with an average fragment length of ~6.2Kb. The K-Means clustering calculated the upper and lower bound fragment size thresholds for the inward read set between 1 bp and 900 bp, and outward read set between 4.2Kb and 8Kb. SVachra_v1.0 was executed using an exclusionary screen out .bed file containing chromosomal centromere and telomere regions (responsible for numerous false positive), a minimum mapping quality threshold of “1”, the unique read mapped flag “-u”, and the clustering quality control heuristic option “-s” that filters overlapping alternate allele breakpoint calls that have lower contributing read counts. For the HS1011 Nextera mate pair data set, SVachra was run using a single node requiring 38Gb of memory and 28 h. SVachra reported a total of 5890 chromosomal aberrations in the HS1011 genome with the following breakpoint annotation type distributions: 57.7% insertions (ranging in size from 10 bp to 1.2 Mb), 17.3% deletions (ranging in size from 10 bp to 1.2 Mb), 4.5% inversions (ranging in size from 14 bp to 6 Mb), 8.2% intra-chromosomal (ranging in size from 64 bp to 223 Mb) and 2.3% inter-chromosomal translocation. The SVachra chromosomal aberrations reported for HS1011 are visualized in Fig. 1c Circos image showing tiled insertions (blue), deletions (red) and inversions (orange), and linked intra-chromosomal (green) and inter-chromosomal (purple) translocations.

**Fig. 2:**
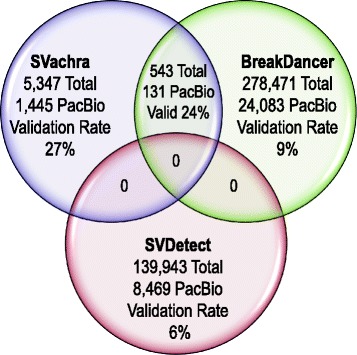
Venn diagram of the reported SVs and Pacific Biosciences validation rates (intersecting mate pairs SVs with PBHoney SVs) for SVachra, BreakDancer and SVDetect

Alternate mate pair specific SV callers BreakDancer [7] and SVDetect [8] were applied to the HS1011 sequencing data for comparison with SVachra. The BreakDancer and SVDetect breakpoint calling programs were executed using recommended parameters for Illumina long insert circularized sequencing libraries. Care was taken to unify input parameters across all programs, unless the comparison tools’ recommended parameters were either stricter or more favorable to their performance (see Additional file 2: S1 for comparison structural variant calling parameters). In addition, a Pacific BioSciences sequencing library of HS1011 (with N50 read lengths of 10Kb) was also sequenced to 20X–coverage for generation of an orthogonal set of validating SVs calls using PBHoney [13]. In order to validate structural variants with the Pacific Biosciences RSII data, we used PBHoney’s ‘force’ procedure. ‘Force’ looks for intra-read discordance or interrupted mapping evidence that match a predicted structural variant in size, location, and orientation in any PacBio read, similar to an egenotyping procedure. A SV call on the HS1011 Nextera data from SVachra, BreakDancer, or SVDetect is considered valid if at least two PacBio reads predict the structural variant with at least 60% reciprocal overlap between the PacBio and mate pair program SVs. Figure 2 shows the total number and overlapping number of SV calls reported by SVachra, BreakDancer and SVDetect and their validation rates when compared to the Pacific BioSciences data. Note that SVDetect did not exhibit any overlapping SV calls with either SVachra or BreakDancer due the fact that the size range of reported SVDetect SVs proved too small (1-9 bp) to exhibit any overlap with SVachra or BreakDancer; although, a limited percent of SVDetect SVs did validate by PacBio. SVachra exhibited the highest validation rate; with 3-fold and 4.5-fold higher PBHoney SV support than Breakdancer and SVDetect, respectively. BreakDancer and SVDetect exhibited higher raw numbers of SVs than SVachra; with 47-fold and 24-fold more breakpoint calls reported. However, Breakdancer and SVDectect reported highly biased sets of HS1011 SV types, with 99.3% of BreakDancer calls being deletions and 100% of SVDetect calls being classified as type “unknown.” In comparison, SVachra reported a diversity of HS1011 SV types with a bias toward insertions, identifying three times more insertions than deletions, with corresponding PacBio validation rates of 26% (INS) and 54% (DEL). From the comprehensive SV ‘Parliament’ discovery set for HS1011, we know that the numbers of insertions and deletions are on the same order of magnitude and that the distribution of deletions exhibits a peak at approximately 300 bp, a characteristic of *Alu* transposon dimorphisms [12]. The loss of deletion detection by SVachra is due to decreased sensitivity for deletions whose length is within the inward facing read fragment size range, which in the case of HS1011 corresponds directly to the high number of expected ~300 bp *Alu* deletions. SVachra’s deletion detection efficiency maybe improved experimentally by ensuring tighter fragment size distributions of the inward and outward facing read types.

**Fig. 3:**
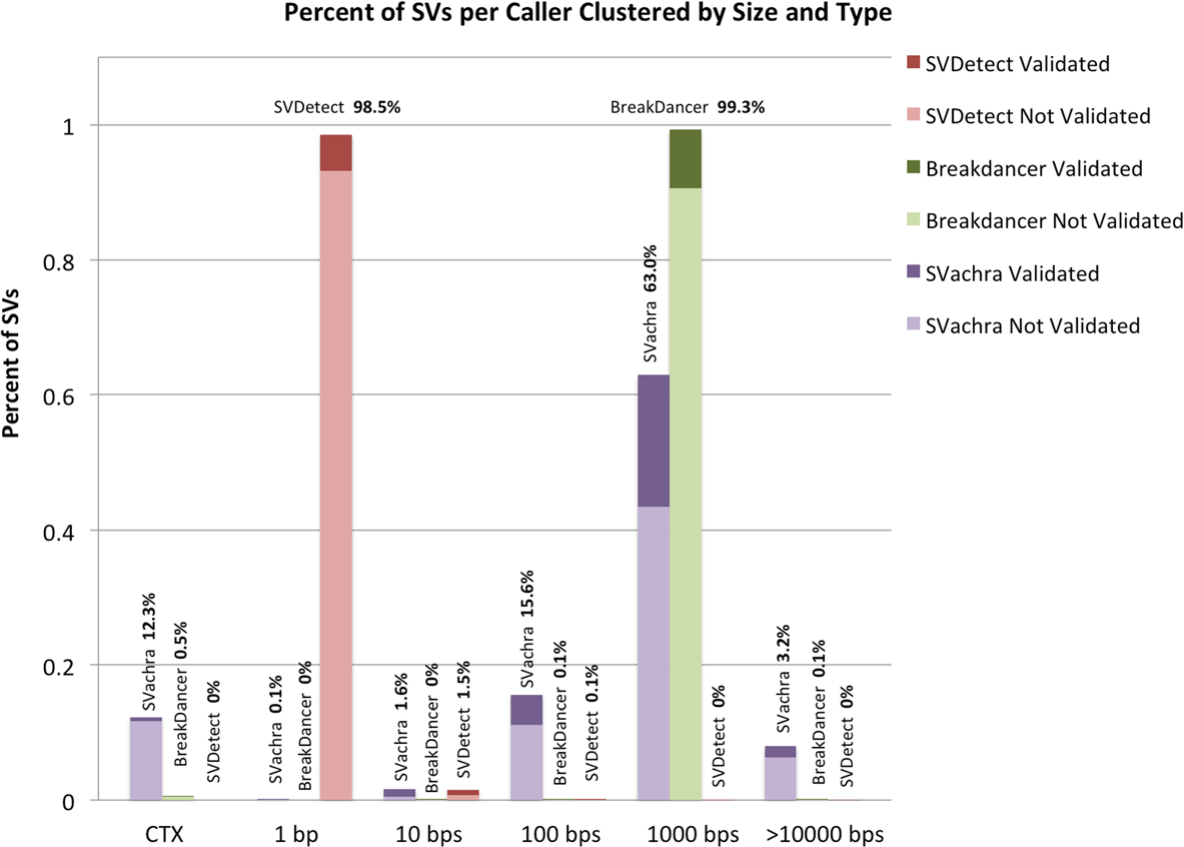
Clustered stacked bar chart of the percent of SVs per mate pair caller. Size clusters are segments per base pair magnitude from zero (CTX) through hundreds of megabases, where UNK, INS, DEL, INV, and ITX types segregate to their annotation sizes above zero. Each stacked bar is color code matched with the Venn diagram, with the top dark region highlighting the validated SVs and the lower lighter region indicating the percent of SVs that did not validate with PBHoney SV calling. The percent of the total reported SVs per bar is indicated

BreakDancer and SVDetect also exhibit SV reporting bias based on putative chromosomal aberration length. Figure 3 shows the percent SVs per caller for the size regimes zero base pairs (inter-chromosomal rearrangement, CTX annotations) through hundreds of megabases. SVDetect reports 98.5% of its putative SVs as having a size between 1 and 9 bp, while BreakDancer reports 99.3% of its putative SVs exhibiting a size range between 100 and 999 bp. SVachra reports the widest distribution of SV size ranges, suggesting a more unbiased SV detection methodology than either BreakDancer or SVDetect which utilize restrictive evaluation areas defined by the empirically calculated insert size distribution [7] or overlapping windows of fixed size [8], respectively.

## Conclusions

The SVachra structural variant discovery tool, which is specific for next-generation mate pair sequencing data, identifies chromosomal aberrations with high specificity across a wide range of variant types and lengths when compared to alternative mate pair sequencing breakpoint calling algorithms. SVachra is designed for whole genome long insert circularized sequencing libraries and scales quadratically on the subset of inconsistently mapped mate pairs based on expected fragment size and orientation. While no built-in parallelization has been implemented, improved time efficiency could be achieved by distributing SV cluster computation across multiple cores, binning inconsistently mapped mate pairs on each possible chromosome pairing relationship. SVachra is unique in its ability to first characterize and then incorporate both inward and outward facing read distributions in mate pair sequencing libraries; providing highly specific breakpoint calling that exhibits a more unbiased detection methodology than alternate mate pair sequencing SV callers.

## Additional files

Additional file 1: SVachra tool I/O descriptions, BG-04-S2. (DOCX 103 kb)

Additional file 2: Supplemental methods, BG-04-S1. (DOCX 139 kb)
