# Supplementary material for: SVachra: a tool to identify genomic structural variation in mate pair sequencing data containing inward and outward facing reads
Source: BMC Genomics. 2017 Oct 3;18(Suppl 6):691. doi: 10.1186/s12864-017-4021-y (PMC5629590; doi:10.1186/s12864-017-4021-y)
Supplement: Supplementary file 1 — SVachra tool I/O descriptions, BG-04-S2. (DOCX 103 kb) [file 12864_2017_4021_MOESM1_ESM.docx]

**SVachra tool input/output descriptions**

SVachra (**S**tructural **V**ariation **A**ssesment of **CHR**omosomal **A**berrations) version 1.0 is a structural variation breakpoint caller, written in ruby, that uses discordant mate pair reads consisting of both inward and outward facing read types; such as the data delivered by Illumina mate pair and Nextera sequencing libraries. The SVachra breakpoint-calling tool utilizes the characteristics of both the inward and outward facing reads to call putative aberrant joins. While inward and outward facing read types inform the assessment of structural variants, reported SVs are orientated to the inward facing read orientations thereby enabling direct comparison and integration of SV call with more common paired end sequencing derived breakpoint calls. SVachra is a play on the word “chakra” and refers to the ability to identify genomic SVs by simultaneously evaluating inward and outward facing mate pairs.

**SVachra requirements**

An installation of samtools as well as the path to the samtools executable is required for SVachra; the latter needs code modification in the SVachra executable pointing to the samtools location. The running of SVachra has been tested using samtools-0.1.18.The following ruby libraries are required for execution:

- getoptlong
- fileutils
- rubygems
- mathn

**SVachra inputs**

Required arguments:

- -f reads.bam

Optional arguments:

- -b screen out .bed file (optional)
- -c minimum number of non-overlapping read pairs to call breakpoint (DEFAULT=2)
- -m minimum mapping quality threshold (DEFAULT=0)
- -n SV annotation name appended with incrementing integer (DEFAULT=SV)
- -u Consider Unique mapping tag (XT:A:U) reads only (optional)
- -s SV cluster quality control filter based on proximity overlaps, filters lower read count alternate allele SVs if present (optional)
- -h help (optional)

**SVachra output**

“Base_Name” equals the bamfile name given by the user.

1. Base_Name.hist.txt - Fragment_Lengths (bins) and Read_Pair_Counts to plot distributions of sequencing library fragment sizes
2. Base_Name.svp - Main Output: listing of all structural variation annotations of types:

- INS – insertion
- DEL – deletion
- INV – inversion
- ITX – intra-chromosomal rearrangement
- CTX – inter-chromosomal rearrangement

Meta-data lines starts with two pound symbols ("##") and describe in human readable format the executed program, abbreviations (“abbrev”), and the key-value attribute descriptions. Following the Meta-Data headers is a column header: “#CHR, OUTERSTART, START, INNERSTART, INNEREND, END, OUTEREND, TYPE, SIZE, INFO.” For any column, a null value is denoted with a period ("."), note that indexing is 0-based.

- Column 1 - chromosome name: Note, we're using chr notation (e.g., "chr1" not just "1")
- Column 2 - outer start: the 5' most boundary of the variant's start - the variant starts downstream of this point (inclusive)
- Column 3 - start: the exact 5' break point of the variant
- Column 4 - inner start: the 3' most boundary of the variant's start - the variant starts upstream of this point (exclusive)
- Column 5 - innner end: the 5' most boundary of the variant's end - the variant ends downstream of this point (inclusive)
- Column 6 - end: the exact 3' break point of the variant
- Column 7 - outer end: the 3' most boundary of the variant's end - the variant ends upstream of this points (exclusive)
- Column 8 - The call from the program
- Column 9 - The size of the variant
- Column 10 - info: your list of KEY=VALUE information provided from your variant caller and documented as described above

1. Base_Name.bed - Bed File of intra-chromosomal rearrangements
2. Base_Name.bedpe - Bed File of inter-chromosomal rearrangements
3. Base_Name.circos.link.txt - Circos link input file for CTX (purple) and ITX (green) annotations
4. Base_Name.circos.tile.txt – Circos tile input file for DEL (red), INS (blue) and INV (orange) annotations
5. Base_Name.lff - lff file format, adapted from the LDAS upload format (<http://www.biodas.org/>) and further described at (http://www.genboree.org/javabin/showHelp.jsp?topic=lffFileFormat). The lff file format is used for SV annotation visualization in Genboree (http://www.genboree.org) and as the input file format for the automated Breakpoint-Primer-Design pipeline (https://github.com/oliverhampton/Breakpoint-Primer-Design).

- Column 1 – Class: A general 'category' for the annotation
- Column 2 – Name: A name for the annotation/annotation group
- Column 3 – Type: The type of SV annotation (INS, DEL, INV, ITX or CTX)
- Column 4 – Subtype: A more specific sub-type for the annotation
- Column 5 – Entry Point: Name of the [entry point](http://www.genboree.org/java-bin/showHelp.jsp?topic=definingAGenome) (e.g. the chromosome) where the annotation is located
- Column 6 – Start: start of the annotation on the entry point
- Column 7 – Stop: end of the annotation on the entry point
- Column 8 – Strand: The orientation of the annotation with respect to the entry point.
- Column 9 – Phase: Whether the annotation is "in-phase" or "out-of-phase" with respect to something, such as the reading frame, or mate-pair read, etc. (currently unimplemented; thus “.” == n/a)
- Column 10 – Score: number of read pairs contributing to the SV call
