# Supplementary material for: SVachra: a tool to identify genomic structural variation in mate pair sequencing data containing inward and outward facing reads
Source: BMC Genomics. 2017 Oct 3;18(Suppl 6):691. doi: 10.1186/s12864-017-4021-y (PMC5629590; doi:10.1186/s12864-017-4021-y)
Supplement: Supplementary file 2 — Supplemental methods, BG-04-S1. (DOCX 139 kb) [file 12864_2017_4021_MOESM2_ESM.docx]

**Supplementary methods**

**Nextera mate pair sequencing library preparation**

The HS1011 Nextera mate pair library was constructed using Illumina Nextera Mate Pair Sample Preparation Kit (Cat. No. FC-132-1001) by following the manufacturer’s Guide (Part # 15035209, Rev.D) with minor modifications. In brief, 3ug of HS1011 genomic DNA was simultaneously fragmented and tagged with a biotinylated mate-pair junction adaptor in transposon-based tagmentation reactions. A total of three reactions (i.e. 1ug genomic DNA per reaction) were set up and incubated at 55°C for 25 minutes. The purified tagmentation products were examined by Agilent 2100 Bioanalyzer using DNA 12000 Chip (cat. no. 5067-1508) for DNA quantification and size distribution. Following the tagmenation procedure, strand displacement was conducted in three reactions. The resulting products were pooled and loaded onto 0.75% agarose gel cassette (Sage Science, Cat. no. BLF7510) for size selection of 6-10Kb fragments using the BluePippin system. Size confirmation was performed for the purified BluePippin product using Agilent 2100 Bioanalyzer. Circularization reaction was conducted using 150ng of the size-selected product, followed by exonuclease treatment to digest any linear molecules left in the circularization reaction. The remaining large circularized DNA fragments were sheared by Covaris S2 device into 700bp fragments and the mate pair fragments containing the biotinylated junction adaptors were purified by binding to streptavidin magnetic beads. Illumina library preparation ensued and consisted of end repair, 3’-end adenylation, paired-end index adaptor ligation and 10 cycles of Ligation-mediated PCR amplification. After validating size and quantification by Agilent 2100 Bioanalyzer, the final Illumina mate-pair library was passed in 10nM concentration aliquot for HiSeq 2500 sequencing. The WGS Illumina Nextera data was generated in 100 x 100 bp mate pair form with an average fragment size of 6.5 Kbp, 71X clone coverage, and approximately 2X read coverage when mapped to the HG19 reference.

**Pacific biosciences large-insert sequencing library preparation**

Large-insert PacBio library preparation was conducted by following the User Bulletin - Guidelines for Preparing 20 kb SMRTbell™ Templates (version 2) and Procedure & Checklist – 20Kb Template Preparation Using BluePippin Size-Selection (version 3) listed in the website (http://www.pacificbiosciences.com/support/pubmap/documentation.html). In brief, a total of 120ug genomic HS1011 DNA was sheared into 20Kb targeted size by using Covaris g-TUBEs (Prod#: 520079, Covaris) on an Eppendorf 5424 centrifuge. Each shearing processed 10ug input DNA and a total of 12 shearings were performed. The sheared genomic DNA was examined by Agilent 2100 Bioanalyzer DNA12000 Chip (Cat. #. 5067-1508, Agilent Technologies Inc.) for size distribution and underwent DNA damage repair/end repair, blunt-end adaptor ligation followed by exonuclease digestion. The purified digestion products were loaded onto pre-cast 0.75% agarose cassettes (Cat.#. BHZ7510, Sage Science) for 7-50Kb size selection using BluePippin Size Selection System (Cat. # BLU0001, Sage Science), and the recovered size-selected library products were purified using 0.5x pre-washed Agencourt AMPure XP beads (A63880, Beckman Coulter). The final libraries were examined by Agilent 2100 Bioanalyzer DNA12000 Chip for size distribution and the library concentrations were determined by Qubit 2.0 Fluorometer (Cat. #. Q32866, Life Technologies).

**Comparison Structural Variant Calling Analysis**

Alternative mate pair specific SV callers were applied to the HS1011 Illumina Nextera sequencing data for comparison with SVachra. BreakDancer and SVDetect programs were executed using recommended parameters for Illumina long insert circularized sequencing libraries. Care was taken to unify comparison tool input parameters with SVachra, unless the comparison tools’ recommended parameters were either stricter or more favorable to performance. The specific input parameters for the comparison SV calling tools are detailed below:

**BreakDancerMax-1.1r112**

BreakDancer was executed using the “-l” option for the analysis of Illumina long insert mate pair libraries, along with a maximum SV size of 1Mb, a minimum mapping quality of 35, a minimum of 2 reads pairs required to establish a breakpoint connection, and a fixed buffer size of 100bp for building putative breakpoint connections. In addition, the insertion and deletion upper and lower bound separation thresholds were set to the empirically calculated mean insert size plus or minus three standard deviations, respectively.

**SVDetect v1.3**

SVDetect was executed using the Illumina mate pair read orientation parameter, the minimum number of pairs in a cluster parameter (nb_pairs_threshold) set to two, and given all the inconsistent Nextera HS1011 read pairs identified by SVachra (SVDetect required the input BAM/SAM file to contain only anomalously mapped read pairs). This ensured that the same set of discordant inward and outward facing mate pair reads, i.e. those read pairs that map with anomalous order, orientation, and/or fragment size, were used for both the SVDetect and SVachra clustering analysis and subsequent SV reporting. SVDetect was also supplied with the HG19 chromosome lengths in a tab delimited text file, as well as, the Nextera forward and reverse read lengths as generated by the Illumina HiSeq 2500 instrument (as specified in SVDetect’s mandatory input parameter list). Finally, SVDetect was supplied with the outward-facing mate pair insert size distribution as calculated by SVachra. The sequencing library insert size distribution is needed by SVDetect for its sliding window size input parameter (window_size=8000bp), a value used to determine the size range of predicted clusters and recommended to be the mean insert size plus two standard deviations from the mean. The sliding window step length (step_length=2000bp) was set to one quarter the window size, the most conservative author recommended value.
